# Is It Safe to Resume Direct Oral Anticoagulants upon Discharge after Hip Fracture Surgery? A Retrospective Study

**DOI:** 10.3390/jcm13010017

**Published:** 2023-12-19

**Authors:** Alona Katzir, Tamar Fisher-Negev, Omer Or, Mahmoud Jammal, Ram Mosheiff, Yoram A. Weil

**Affiliations:** Department of Orthopedic Surgery, Hadassah Medical Organization and Faculty of Medicine, Hebrew University of Jerusalem, Kalman Yaakov Man Street, Jerusalem 9112001, Israel; alonakat@gmail.com (A.K.); or@hadassah.org.il (O.O.); jammalm@hadassah.org.il (M.J.); ramim@hadassah.org.il (R.M.); weily@hadassah.org.il (Y.A.W.)

**Keywords:** reoperation, hip fracture, anticoagulants, LMWH, internal fixation, hemiarthroplasty

## Abstract

This study aimed to examine the incidence rate of early reoperations following hip fracture surgery and determine the safety of resuming direct oral anticoagulants. Many orthopedic surgeons are reluctant to resume chronic anticoagulation therapy for patients after surgical intervention for hip fractures. One of the main reasons is the potential for reoperation in the case of surgical complications. We conducted a retrospective cohort study at an Academic Level I trauma center, reviewing the records of 425 geriatric patients (age > 60) who underwent hip fracture surgery between 2018 and 2020, including a subgroup treated with direct oral anticoagulants prior to hospitalization. The study assessed the incidence rate of complications requiring early reoperation. Out of the 425 patients, only nine (2%) required reoperation within a month after discharge, with two (0.5%) on chronic anticoagulation therapy. None of the reoperations were urgent, and all were performed at least 24 h after re-admission. The findings revealed a very low incidence rate of reoperations in patients who underwent hip fracture surgery, with no reoperations performed within 24 h of re-admission. Consequently, we believe that resuming chronic direct oral anticoagulants is a safe and effective approach when discharging patients after hip fracture surgery.

## 1. Introduction

Proximal femoral fractures are common in the geriatric population, and they impose a long-lasting physical and financial burden [1]. The one-month postoperative mortality rate is around 10%, and one-year mortality following hip fractures ranges between 15–33% [2,3,4,5,6]. Numerous studies have provided evidence regarding the crucial role of early surgical intervention and mobilization in preventing severe medical complications including venous thromboembolic events (VTE), and in reducing mortality [7]. In the United States, the current standard protocol is to operate on proximal femur fractures within 48 h post-admission. In the United Kingdom, this timeframe is 36 h [8]. Pincus et al. conducted a study recommending early surgical intervention, demonstrating a significant reduction in postoperative complications for patients who underwent surgery within 24 h, and even within 12 h [9,10]. These findings highlight the potential benefits of prompt surgical treatment for proximal femur fractures in terms of improving outcomes and reducing complications.

A significant portion of geriatric patients with low-energy hip fractures receive chronic anticoagulation (AC) therapy as part of their treatment [11,12,13,14]. AC may include various medications, such as direct oral anticoagulants (DOACs), warfarin, or low-molecular-weight heparin (LMWH). However, the optimal approach to perioperative management for patients who may require heparin bridging, are on DOACs, or take multiple antiplatelet drugs remains uncertain [15]. In such cases, a customized anticoagulation treatment approach may be more appropriate and beneficial.

Previous studies indicated increased rates of AC therapy in the geriatric population who are candidates for emergent or elective surgery [16]. For decades it was a common practice to withhold DOACs or warfarin in the perioperative period, as well as in the immediate postoperative period, usually for 14–30 days [17]. LMWH is administered for the immediate and early (3–6 weeks) postoperative period based on well-studied and established venous thromboembolism protocols [18,19,20]. The rationale for switching from DOACs to LMWH therapy is derived from long experience with LMWH in postoperative VTE prophylaxis and from the different pharmacokinetics of these medications. As LMWH has a shorter half-life and is excreted faster than DOACs or warfarin, it is thought to be advantageous when urgent reoperation is required or if significant bleeding occurs [21,22].

In practice, however, full adherence to LMWH can be challenging, and many patients discontinue the treatment prematurely [23,24,25,26]. In addition, the exact duration of VTE prophylaxis following hip fracture surgery is still unknown and debatable [27,28].

The objective of this study is to determine the incidence rate of early reoperations following hip fracture surgery and to evaluate whether resuming DOACs in the immediate postoperative period would affect the safety of early reoperation, when needed. Our hypothesis was that the renewal of DOACs would not risk patients, as urgent reoperations are uncommon and the benefits of resuming DOACs outweigh the risks.

## 2. Methods

This is a retrospective blinded cohort study in an Academic Level I trauma center with an Institutional Review Board approval. Data were extracted from the hospital’s electronic medical records. The inclusion criteria were all patients older than 60 that were hospitalized in our center with a hip fracture between 1 April 2018 to 31 March 2020. Our data collection stopped on 31 March 2020, as our postoperative AC treatment protocol changed following the data collected in this study.

A cephallomedullary nail (CMN) was used for all intertrochanteric fractures. Cannulate cancellous screws were used to fix non-displaced femoral neck fractures. Displaced femoral neck fractures in this population were treated with cemented bipolar hemiarthroplasty. All cases were operated under the supervision of fellowship-trained trauma surgeons. Exclusion criteria were pathological fractures, high-energy trauma, patients who were taking warfarin, and intra-cranial or other severe bleeding precluding the use of AC therapy. We examined the total incidence of complications requiring reoperations during the month following the index operation. We also examined the rate of postoperative complications for patients on chronic AC therapy. It should be noted that patients who were taking warfarin routinely prior to their surgery were discharged with LMWH injections, since resuming warfarin is not trivial and was conducted by the primary healthcare physician.

Other parameters extracted from the patient’s medical files were gender, age, and types of anesthesia.

## 3. Results

We identified 450 patients over the age of 60 who were admitted with low-energy hip fractures during the study period. Out of those, 425 patients were operated on. Twenty-five low-demand non-ambulatory patients with sub-capital fractures were treated with intracapsular injectable pain relievers and were not operated on. One-month postoperative mortality for all patients in cohort was 4.2% (18 patients).

Two-thirds of the patients in this study were females. The mean age was 80 (±9.6) and about half of the patients older than 80 (Table 1). 75% of the patients were treated with internal fixation (IF) and 25% with hemiarthroplasty (HA). About 17% (69/407) of the patients were on chronic AC therapy preoperatively (18% of the patients treated with CMN and 13% treated with HA).

**Figure 1:**
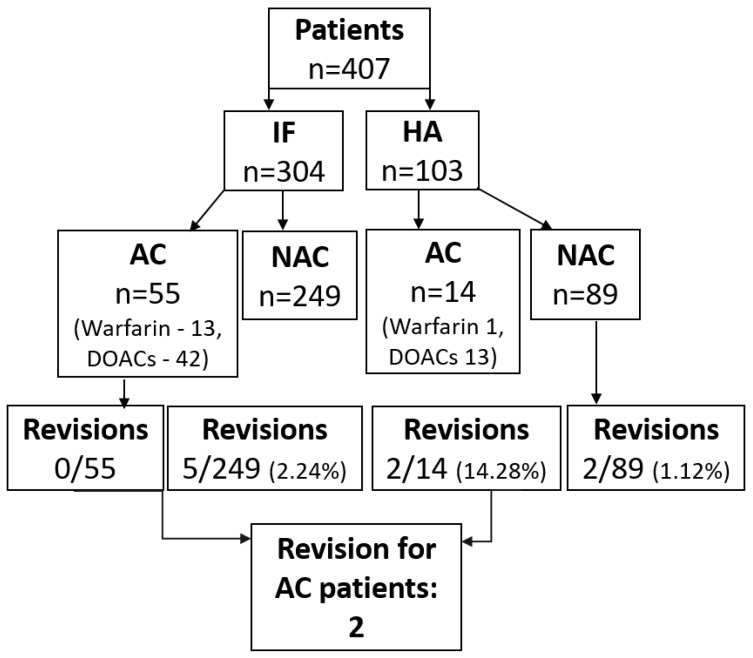
Flow chart of patients’ surgical treatment and anticoagulation treatment: **IF**—internal fixation, **HA**—hemiarthroplasty, **AC**—anticoagulation **NAC**—no anticoagulation.

The total reoperation rate in this study was 2.2% (9/407), and two out of the nine patients that underwent reoperation were on prior AC therapy (Figure 1).

The mean time from index surgery to reoperation was 17.6 days (7–30 days, Table 2) and seven out of nine patients who were reoperated on were older than 80.

Five of the nine patients that needed reoperation were after IF with CMN, and the remaining four underwent closed reduction after a dislocation of hemiarthroplasty (Table 3). Common complications after surgeries include infections, dislocations, fractures, nonunions, malunions, and loss of fixation. Infections can be superficial wound infections or deep surgical site infections that are usually harder to treat. Fractures might occur around the fixation device or implants in the case of hemiarthroplasties. Cutout refers to a loss of fixation in the case of IF with a CMN.

To our knowledge, the patients on chronic AC therapy in our study did not have any thromboembolic events or major bleedings that necessitated any intervention during the month following discharge. As for the patients without chronic AC therapy, only two had VTE the month following surgery, one with a new DVT and one with a PE. Chronic AC therapy with DOACs was initiated for both.

As mentioned, 18 out of the 425 patients in our cohort died within a month after surgery. The one-month postoperative mortality rate in well-established literature is around 10% for this type of surgery. We were unable to determine the exact causes of these deaths, as most of those patients died in their homes after discharge. However, hip fractures are not innocent fractures and usually represent general deterioration with high mortality rates. Mortality rates are much higher without surgery. We understand that some of those patients might have had strokes/VTE or bleeding.

## 4. Discussion

The main finding of this study revealed a notably low reoperation rate within the initial month following proximal femoral fractures, which is consistent with other studies [29,30,31]. The study population predominantly consisted of individuals aged 80 years and older, with ASA scores ranging between 2 and 3E. These characteristics align with the data collected in previous studies [32,33,34,35]. While there appears to be a correlation between advanced age, higher ASA scores, and reoperations [36,37], it is important to note that reoperations remained infrequent. None of the patients requiring reoperation were operated on urgently. Most of the patients presented with dislocation after hemiarthroplasty and underwent closed reduction. Out of the 425 patients enrolled in this study, 69 were receiving chronic anticoagulation therapy, and only two of them required closed reduction for a dislocated hemiarthroplasty. This procedure was found to be safe, even in fully anticoagulated patients [38]. In our study, we observed that none of the patients with AC therapy who underwent IF needed early reoperation. Out of the other 356 patients who underwent surgery for hip fractures without prior anticoagulation therapy, seven required reoperation. Two patients who underwent hemiarthroplasty required closed reduction, while five patients who underwent IF required surgery due to other complications–infection, cutout of an implanted device, dislocation of hemiarthroplasty, and distal femur fracture. All the reoperations in our study were non-urgent, and they were conducted at least 24 h after admission, providing ample time for patient preparation before the surgery. Consequently, considering oral AC therapy as a postoperative preventive measure could be a safe choice for all patients [39].

Traditionally, our department, like many others worldwide, converted chronic AC therapy patients to LMWH after surgery, with a recommendation for LMWH treatment for the first month post-surgery. This practice aimed to enhance safety in case of reoperation. Unfortunately, adherence of patients to LMWH treatment after surgery is low. Wilke et al. in 2010, found 13–21% non-adherence to LMWH in outpatients’ post-major orthopedic surgery, missing about 38–43% of their injections [24]. There are various reasons for poor compliance, such as financial burden; inconvenient administration; pain and possible hematoma in the injection site; or even a simple injection phobia. Due to the difficulties that arise from the need for self-injection ambulatory, some experts recommend nursing service involvement in cases of outpatient non-compliance risk [40,41]. Although DOACs seem to be a more practical treatment for outpatients, many physicians are reluctant to renew DOACs therapy, which could force them to delay an intervention in the case of postoperative complications, which might require urgent surgical intervention [22,42]. Considering the potential complications, such as infection, nonunion or malunion, implant cutout, and periprosthetic fracture, it should be feasible to prepare chronic AC patients for reoperation without significant issues. In addition, emerging evidence suggests that delaying hip fracture surgery in patients on DOACs does not lead to improved outcomes in terms of transfusion rates, and that the bleeding risk with DOACs is not higher than with LMWH [43]. With the established safety profile of DOACs and our results showing a minimal chance of reoperation, resuming oral anticoagulants after surgery may be a safe and practical option for patients on chronic AC. Patients who were on oral anticoagulants preoperatively find it easier to return to their prior AC regimen after surgery, likely resulting in better adherence rates [24,25,26]. Moreover, we should consider treating patients after hip fracture surgery with oral anticoagulation therapy instead of LMWH, even in cases where patients were not previously on AC therapy. Overall, the available evidence suggests that DOACs are a viable and effective alternative to LMWH, offering comparable efficacy in VTE prevention with certain advantages, particularly in terms of bleeding risks and convenience of oral administration [15,44].

Over the years, there has been a search for an optimal approach to venous thromboembolism (VTE) prophylaxis following major orthopedic procedures. DOACs have demonstrated comparable safety and effectiveness to LMWH in VTE prophylaxis for hip and knee arthroplasty. Nederpelt et al. found no differences in VTE prevalence between patients receiving DOACs and those receiving LMWH [39]. The METRIC study group suggested that twice-daily aspirin might not be inferior to twice-daily LMWH in VTE prophylaxis after fracture surgery, although this was observed in a younger population [45]. Recently, new perioperative guidelines for antithrombotic therapy were published [15], recommending the cessation of DOACs between 1 and 4 days prior to surgery or procedures, based on the specific DOAC, patient factors (e.g., chronic kidney disease), individual patient bleeding risk, and the anticipated surgical or procedural bleeding risk. DOACs can be resumed safely 24 to 72 h after surgery. This literature supports our hypothesis that administration of DOACs after surgery would not risk patients and that DOACs are not inferior to LMWH.

We acknowledge that our study has certain limitations. Our study is retrospective, with a relatively small number of patients on chronic AC therapy and a limited number of complications. While reoperations are rare, there may still be cases in which a relatively urgent intervention is required. In addition, all surgeries in our study were performed by or under the supervision of a fellowship-trained trauma surgeon, the case might be different in other establishments. As mentioned in our results, after March 2020 our department changed the AC treatment protocol. After observing the low incidence rate of reoperations, we decided to resume chronic AC therapy after index surgery for all patients with known indications and hip fractures. Furthermore, we were unable to determine the exact reason of death for the 18 patients who died the month after surgery. We understand that some of those patients might have had strokes/VTE or bleeding.

In conclusion, the incidence of early reoperations in patients on chronic AC therapy after hip fracture surgery is very low. Typically, no urgent intervention is necessary for these patients. Therefore, it is safe to resume chronic AC therapy upon discharge after hip fracture surgery. We should also consider the initiation of oral AC therapy for all patients after hip fracture surgery as a preventative measure, rather than using LMWH.

## Figures and Tables

**Table 1:** Demographics of the study population.

		IF (*n* = 304)	HA (*n* = 103)
Gender	**Females**	193	57
Age	** 80≥ **	155	54
	** 80< **	149	49
Anesthesia	**General**	235	83
	**Spinal**	69	20
Chronic AC	**YES**	55	14
ASA	**1**	7	1
	**1E**	1	0
	** 2 **	99	5
	**2E**	29	30
	**3**	94	37
	**3E**	53	19
	**4**	9	8
	**4E**	12	3

**IF**—internal fixation, **HA**—hemiarthroplasty, **AC**—anticoagulation, **ASA** (American Society of Anesthesiologists)—1–4E scale for physical status classification ex. ASA1—normal healthy, ASA2—mild systemic disease, ASA3—severe systemic disease, ASA4—severe systemic disease threatening patient’s life.

**Table 2:** Demographics, index surgery, and complications of reoperated patients.

Age	Gender	Surgery	AC	Days after Discharge	ASA	Anesthesia	Complication
71	Female	IF	No	14	3	General	Infection
74	Female	IF	No	16	2	General	Distal femur fracture
80	Male	HA	No	7	2	General	Dislocation
80	Male	HA	Yes	7	2	General	Dislocation
81	Female	HA	Yes	30	3	Spinal	Dislocation
87	Female	IF	No	21	2	General	Cutout
89	Male	HA	No	30	3E	General	Dislocation
90	Female	IF	No	17	3	General	Cutout
91	Female	IF	No	17	2	General	Cutout

**Table 3:** Demographics and surgical treatment of patients who died within a month following surgery.

		IF (*n* = 16)	HA (*n* = 2)
Gender	**Females**	11	1
Age	**≤80**	4	
	**>80**	12	2
Anesthesia	**General**	12	1
	**Spinal**	4	1
Chronic AC	**YES**	4	
ASA	** 2 **	1	
	**2E**	1	
	**3**	6	2
	**3E**	1	
	**4**	3	
	**4E**	4	
Death relative to hospitalization	**During/right after**	16	1
	**After**	0	1

## Data Availability

The data presented in this study are available on request from the corresponding author.
